# When a Seizure Is Not Just a Seizure: Rhabdomyolysis and Acute Kidney Injury as a Reminder to Check Creatine Kinase

**DOI:** 10.7759/cureus.105649

**Published:** 2026-03-22

**Authors:** Ahmed S Alsherbeeny, Latif Rahman, Loay T Soliman, Husain Anwar

**Affiliations:** 1 Acute Medicine, University Hospitals of Leicester NHS Trust, Leicester, GBR; 2 Cardiology, Cairo University, Cairo, EGY; 3 Emergency Medicine, University Hospitals of Leicester NHS Trust, Leicester, GBR

**Keywords:** acute kidney injury, creatine kinase, generalized tonic clonic seizure, muscle injury, pigment nephropathy, rhabdomyolysis

## Abstract

Generalized tonic-clonic seizures are a recognized cause of rhabdomyolysis and may be complicated by acute kidney injury, particularly when muscle injury is severe or prolonged. We describe the case of a 53-year-old man who presented following a witnessed generalized seizure and collapse, with subsequent development of severe rhabdomyolysis and stage three acute kidney injury. Initial assessment focused on neurological and infectious causes of altered consciousness, including neuroimaging and cerebrospinal fluid analysis, which did not demonstrate an acute intracranial or central nervous system infectious process. During admission, renal function progressively deteriorated, accompanied by electrolyte abnormalities. Serial biochemical testing later revealed a marked rise in creatine kinase, peaking at greater than 89,000 international units per liter, confirming severe rhabdomyolysis. Extensive investigations excluded alternative causes of renal impairment, including autoimmune, vasculitic, obstructive, and glomerular pathology. The patient was managed with aggressive intravenous fluid resuscitation, electrolyte correction, and supportive care, resulting in gradual improvement in renal function and down-trending creatine kinase levels without the need for renal replacement therapy. This case highlights the evolving nature of seizure-associated rhabdomyolysis and its potential to cause significant acute kidney injury. It emphasizes the importance of considering rhabdomyolysis in patients presenting after generalized seizures and supports early measurement and monitoring of creatine kinase to guide timely management and reduce the risk of renal complications.

## Introduction

Rhabdomyolysis is a clinical syndrome caused by acute skeletal muscle injury leading to the release of intracellular contents into the circulation, including creatine kinase (CK) and myoglobin [1]. Although traumatic injury and drug-related toxicity are common precipitants, generalized tonic-clonic seizures are a well-recognized non-traumatic cause, resulting from intense muscle contraction and, in some cases, prolonged immobilization during the postictal period [2,3]. The clinical presentation is often variable, and classical features such as myalgia or dark-colored urine may be absent, particularly in patients presenting with altered consciousness or competing diagnostic priorities [1].

Acute kidney injury (AKI) represents one of the most serious complications of rhabdomyolysis and occurs in a substantial proportion of affected patients [1,4]. The pathogenesis is multifactorial and includes myoglobin-mediated tubular toxicity, intraluminal cast formation, and renal vasoconstriction, all of which may be exacerbated by hypovolemia, sepsis, or metabolic derangement [4]. Importantly, creatine kinase levels typically rise over the first 24 to 72 hours following muscle injury and may peak several days after the inciting event, highlighting the importance of early assessment and serial biochemical monitoring in at-risk patients [1,5].

In patients presenting after a generalized seizure, initial evaluation often focuses on exclusion of intracranial pathology or central nervous system infection, which may delay recognition of systemic complications such as rhabdomyolysis [3]. We report a case of seizure-associated rhabdomyolysis complicated by severe acute kidney injury, in which creatine kinase rose markedly during admission. This case illustrates the evolving biochemical profile of rhabdomyolysis following seizures and reinforces the importance of considering early measurement and follow-up of creatine kinase and renal function in patients presenting after generalized seizures, particularly when reduced consciousness or prolonged collapse is suspected.

The educational value of this case lies in the delayed biochemical evolution of rhabdomyolysis and acute kidney injury despite initially non-severe renal indices, reinforcing the importance of early suspicion and repeat testing after generalized seizures.

## Case presentation

A 53-year-old man was admitted following a generalized tonic-clonic seizure with a subsequent reduced level of consciousness. The patient's height was 165 cm, weight 56 kg, with a body mass index of 19.5 kg/m². He had no prior history of chronic kidney disease. There was no reported prolonged immobilization, excessive exertion, trauma, illicit drug use, or exposure to known myotoxic agents. On admission, he was hemodynamically stable. Initial laboratory investigations demonstrated preserved renal function, with a serum creatinine of 108 µmol/L, urea of 2.4 mmol/L, and estimated glomerular filtration rate (eGFR) of 67 mL/min/1.73 m². Creatine kinase (CK) was not measured at presentation. Serial biochemical testing, including repeated measurements of creatine kinase and renal function, was subsequently performed to monitor the biochemical evolution of rhabdomyolysis during hospitalization.

Development of acute kidney injury

Within 72 hours of admission, the patient developed rapidly progressive acute kidney injury (AKI). By hospital day three, serum creatinine had increased markedly to 482 µmol/L, urea to 11.8 mmol/L, and eGFR declined to 11 mL/min/1.73 m², meeting criteria for Kidney Disease: Improving Global Outcomes (KDIGO) stage 3 AKI [1]. At this stage, CK was measured and found to be 89,345 U/L, consistent with severe rhabdomyolysis [2]. Renal dysfunction persisted on day four, with creatinine 314 µmol/L, urea 11.9 mmol/L, and eGFR 18 mL/min/1.73 m², while CK remained markedly elevated at 88,808 U/L, indicating ongoing muscle injury (Table 1).

**Table 1:** Temporal trend of renal function and creatine kinase during hospitalization. This table demonstrates the temporal relationship between seizure, severe rhabdomyolysis, and acute kidney injury, followed by biochemical recovery. Creatine kinase was not measured on day one. eGFR values reported as “>90 mL/min/1.73 m²” reflect recovery to normal renal function.

Day	Creatinine (umol/L)	Reference range	Urea (mmol/L)	Reference range	eGFR (mL/min/1.73 m^2^)	Reference range	Creatine kinase (U/L)	Reference range
1	108	60-105 umol/L	2.4	2.5-7.8 mmol/L	67	>90 ml/min/1.73 m^2^		40-320 IU/L
3	482		11.8		11		89345	
4	314		11.9		18		88808	
5	154		10.4		43		65983	
6	78		6.3		>90		26828	
7	72		4.7		>90		9905	

Biochemical recovery

**Figure 1:**
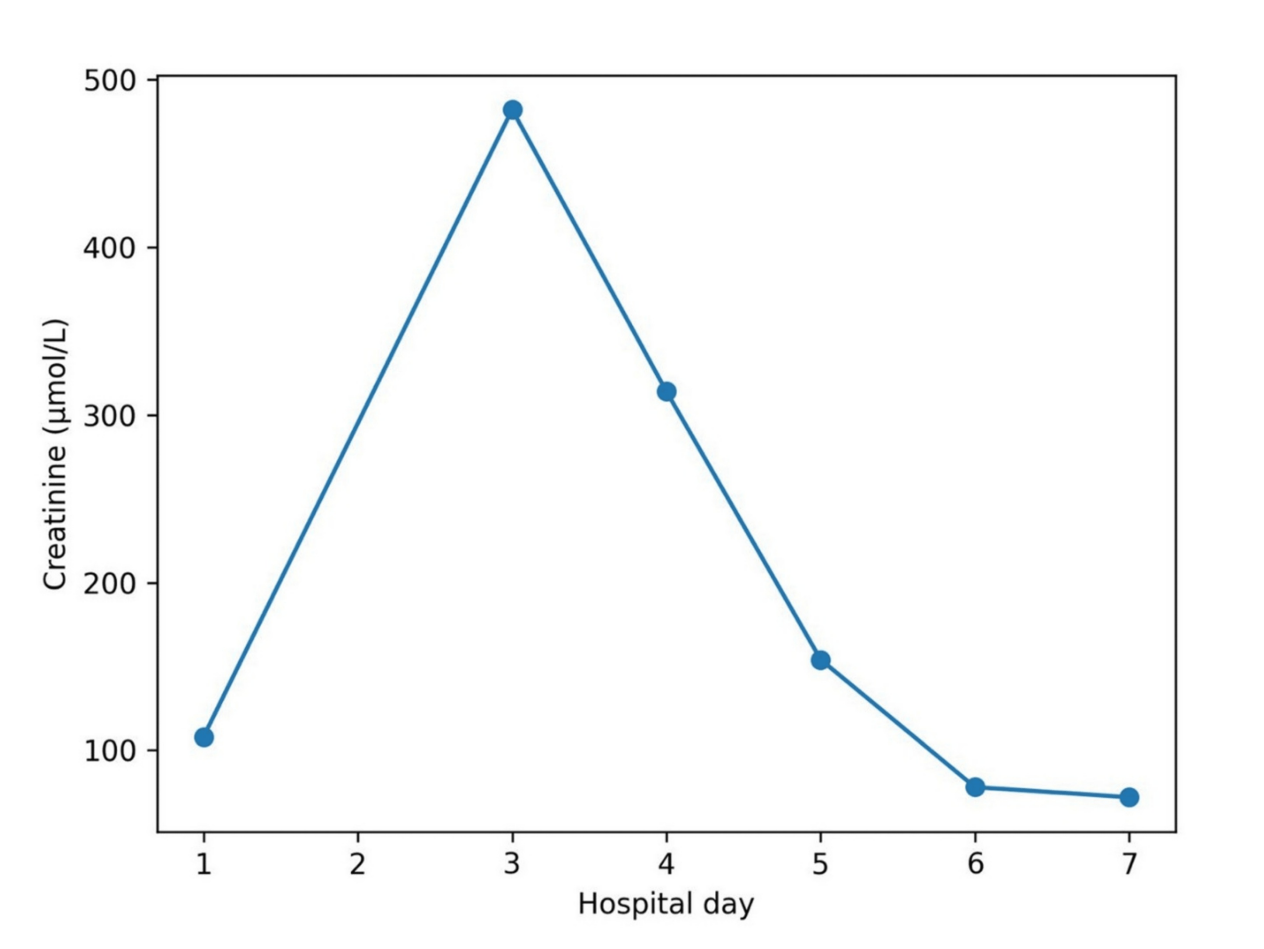
Trend of serum creatinine during hospitalization. This figure demonstrates the rapid rise in serum creatinine following seizure, consistent with acute kidney injury, followed by gradual recovery over subsequent days.

**Figure 2:**
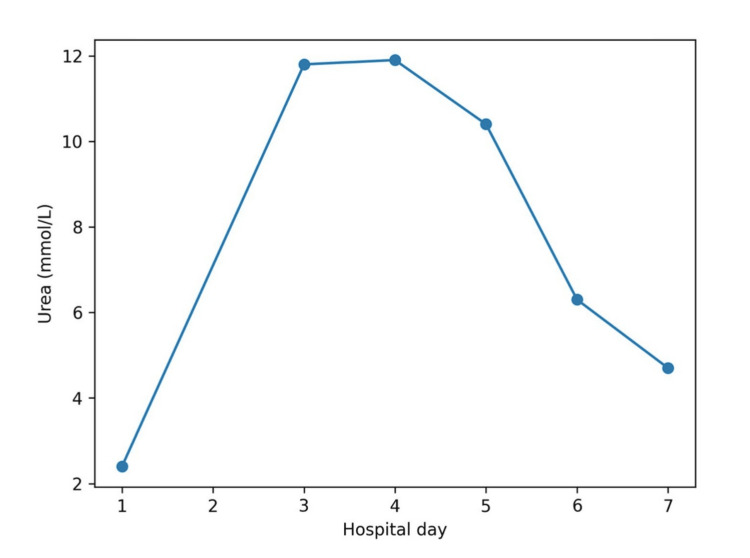
Trend of serum urea during hospitalization. Serum urea increased in parallel with worsening renal function and subsequently declined as kidney function recovered.

**Figure 3:**
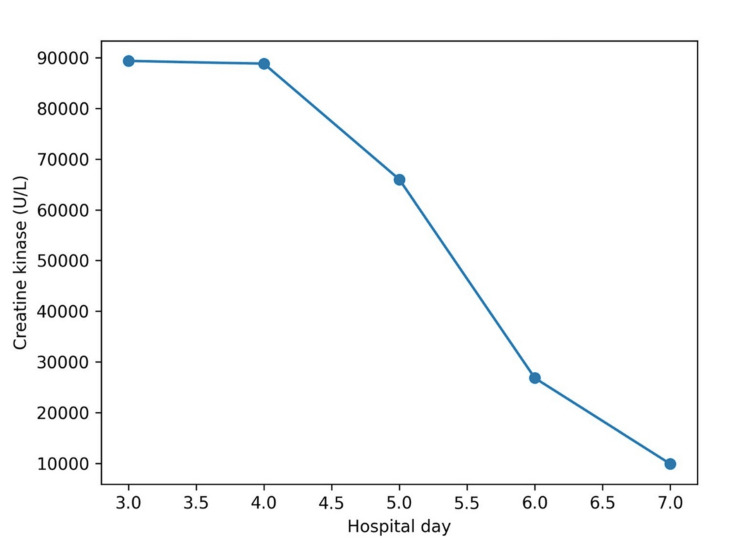
Trend of serum creatine kinase following generalized seizure. This figure illustrates the marked elevation and gradual decline of serum creatine kinase following a generalized tonic-clonic seizure. Creatine kinase values (U/L) are plotted against hospital day, with individual data points corresponding to measured values: day three (89,345 U/L), day four (88,808 U/L), day five (65,983 U/L), day six (26,828 U/L), and day seven (9,905 U/L). Creatine kinase was not measured on day one. The delayed peak and gradual decline are consistent with severe rhabdomyolysis.

As illustrated in Figures 1-3, from day five onward, with appropriate intravenous fluid therapy, renal function improved in parallel with a gradual decline in CK. Serum creatinine decreased to 154 µmol/L on day five, 78 µmol/L on day six, and 72 µmol/L on day seven, with corresponding improvement in urea and normalization of eGFR (>90 mL/min/1.73 m²). CK levels declined more slowly, remaining significantly elevated on day five (65,983 U/L) before decreasing to 26,828 U/L on day six and 9,905 U/L on day seven, consistent with the known delayed clearance of CK following muscle injury [2,3].

Additional investigations

Given the severity of acute kidney injury, further laboratory investigations were performed to exclude alternative intrinsic renal pathologies. Complement levels, autoimmune serology, serum protein electrophoresis, immunoglobulin profile, and relevant infectious markers are summarized in Table 2. Complement concentrations were within reference ranges. Autoimmune screening, including antinuclear antibody, anti-glomerular basement membrane antibody, proteinase 3, and myeloperoxidase antibodies, did not support a diagnosis of immune-mediated glomerulonephritis or systemic vasculitis. Serum protein electrophoresis demonstrated no paraprotein. Immunoglobulin G was reduced, while immunoglobulin A and M were within reference limits. Anti-streptolysin O titer was not elevated. Collectively, these findings did not indicate an alternative immune, vasculitic, or paraproteinemic cause of renal impairment.

**Table 2:** Renal autoimmune and immunological workup in severe acute kidney injury. Laboratory investigations were undertaken to exclude alternative intrinsic renal pathologies in the setting of severe acute kidney injury. Results are presented with corresponding units, laboratory reference ranges, and clinical interpretation.

Investigation	Result	Units	Reference range	Interpretation
Complement C3	1.00	g/L	0.90–1.80	Within reference range
Complement C4	0.27	g/L	0.10–0.40	Within reference range
Antineutrophil cytoplasmic antibody (ANCA) (immunofluorescence)	Indeterminate	-	Negative	Indeterminate
Proteinase 3 antibody (PR3)	Negative	-	Negative	Negative
Myeloperoxidase antibody (MPO)	Negative	-	Negative	Negative
Anti-glomerular basement membrane antibody	13.1	CU	0–19.9	Negative
Antinuclear antibody (ANA)	Negative	-	Negative	Negative
Serum protein electrophoresis	No paraprotein detected	-	No paraprotein	Normal
Immunoglobulin G	5.1	g/L	7.0–16.0	Low
Immunoglobulin A	Within reference range	g/L	0.70–4.00	Normal
Immunoglobulin M	Within reference range	g/L	0.40–2.30	Normal
Anti-streptolysin O titer	<200	IU/mL	<200	No evidence of recent streptococcal infection

Clinical interpretation

The temporal association between generalized seizure, delayed but marked elevation of creatine kinase, severe but reversible AKI, and subsequent parallel biochemical recovery strongly supports a diagnosis of seizure-associated rhabdomyolysis complicated by acute kidney injury. This case highlights the importance of early measurement and serial monitoring of creatine kinase in patients presenting after generalized seizures, even when initial renal function appears preserved [2-4].

## Discussion

Rhabdomyolysis is a well-established systemic complication of generalized tonic-clonic seizures and represents an important, potentially reversible cause of acute kidney injury (AKI) [1,2]. The condition results from sustained, forceful skeletal muscle contraction leading to myocyte disruption and release of intracellular contents, including creatine kinase (CK) and myoglobin, into the circulation [2,3]. While trauma, exertion, and drug toxicity are commonly recognized precipitants, seizures alone are sufficient to cause severe rhabdomyolysis and subsequent renal injury, even in the absence of external muscle trauma or prolonged immobilization [4,5].

This case demonstrates a characteristic but clinically hazardous temporal pattern in which renal function is initially preserved following a generalized seizure, followed by the delayed onset of severe AKI. On admission, the patient had near-baseline renal indices, which may falsely reassure clinicians and delay further investigation. However, within 72 hours, renal function deteriorated rapidly to KDIGO stage 3 AKI, coinciding with the first documented measurement of markedly elevated CK (>89,000 U/L). This delayed biochemical evolution is well described in the literature and reflects the time course of muscle breakdown, systemic absorption of myoglobin, and secondary renal tubular injury [3,6].

Creatine kinase levels in seizure-associated rhabdomyolysis typically peak between 48 and 72 hours after the inciting event, rather than at initial presentation [2,6,7]. In this case, CK was not measured at admission and was only identified after the onset of significant renal dysfunction. Early clinical assessment focused on stabilization and exclusion of acute neurological or infectious causes of the seizure. However, this case illustrates how rhabdomyolysis may evolve with delayed biochemical elevation, emphasizing the importance of early and repeated creatine kinase measurement following generalized seizures. The magnitude of CK elevation observed greatly exceeded thresholds previously associated with increased risk of renal failure. Large cohort studies have consistently shown that CK levels above 5,000 U/L, and particularly above 15,000 U/L, are strongly predictive of AKI severity and adverse renal outcomes [8,9]. Despite CK values exceeding 80,000 U/L in this patient, renal recovery occurred with supportive management alone, highlighting the potential reversibility of pigment-induced AKI when recognized and treated appropriately.

The pathogenesis of AKI in rhabdomyolysis is multifactorial. Myoglobin-mediated oxidative injury, intrarenal vasoconstriction, tubular obstruction by pigment casts, and volume depletion all contribute to renal impairment [3,10]. The parallel improvement in creatinine, urea, and estimated glomerular filtration rate with declining CK levels in this case supports a direct causal relationship between muscle injury and renal dysfunction. Importantly, the slower normalization of CK compared with renal indices is consistent with prior observations and reflects ongoing clearance of muscle enzymes after renal recovery has begun [6].

Alternative causes of intrinsic renal disease were systematically excluded. Normal complement levels, negative autoimmune serology, absence of paraproteinemia, and lack of clinical features suggestive of glomerulonephritis or vasculitis argue against immune-mediated renal pathology. The temporal association between seizure activity, delayed CK elevation, and AKI, combined with biochemical recovery following conservative therapy, strongly supports rhabdomyolysis as the primary etiology of renal injury in this case.

Compared with previously published case reports and observational studies, this case is notable for the severity of biochemical derangement, delayed recognition of rhabdomyolysis, and complete renal recovery without the need for renal replacement therapy [5,7]. It reinforces existing recommendations that generalized seizures should prompt early and serial measurement of CK and renal function, even when initial laboratory results are reassuring and classical symptoms such as myalgia or dark urine are absent [2,4].

In clinical practice, seizures are often approached as isolated neurological events. This case emphasizes that they should instead be regarded as systemic insults with the potential for significant extracerebral complications. Early recognition of seizure-associated rhabdomyolysis allows timely initiation of supportive measures, including aggressive hydration, avoidance of nephrotoxins, and close biochemical monitoring, thereby reducing renal morbidity and improving outcomes.

The principal practical lesson from this case is that initially non-severe renal indices do not exclude evolving seizure-associated rhabdomyolysis, and repeat biochemical assessment may be warranted when clinical suspicion persists.

## Conclusions

Seizure-associated rhabdomyolysis is a recognized but potentially under-recognized cause of acute kidney injury following generalized tonic-clonic seizures. This case illustrates how both creatine kinase elevation and renal impairment may evolve in a delayed fashion despite initially reassuring laboratory findings. Early measurement and serial monitoring of creatine kinase and renal function should therefore be considered in patients presenting after generalized seizures, even in the absence of classical symptoms of rhabdomyolysis. Prompt recognition and supportive management may facilitate renal recovery and reduce the risk of severe complications.
